# Inversion Motion of Xanthene and Detection of Its Oxidation Product Xanthone from Gas-Phase Rotational Spectroscopy

**DOI:** 10.3390/molecules30132801

**Published:** 2025-06-29

**Authors:** Celina Bermúdez, Manuel Goubet, Elias M. Neeman

**Affiliations:** 1 Departamento de Química Física y Química Inorgánica, Facultad de Ciencias—I.U. CINQUIMA, Universidad de Valladolid, Paseo de Belén 7, 47011 Valladolid, Spain; 2 University of Lille, CNRS, UMR 8523—PhLAM—Physique des Lasers Atomes et Molécules, F-59000 Lille, France

**Keywords:** rotational spectroscopy, large-amplitude motion, oxidation effect

## Abstract

The rotational spectra of xanthene and its oxidation product xanthone were investigated by combining quantum chemical calculations with Fourier transform microwave spectroscopy in a jet-cooled environment. Xanthone was unexpectedly generated in the experiment when water was present in the reservoir of xanthene leading to the total disappearance of xanthene after few hours. Structurally, xanthone shows a near planar disposition, whereas xanthene exhibits a non-planar geometry with both benzene rings twisted out of the molecular plane. This geometry enables an inversion motion between two equivalent conformers, giving rise to a splitting in the ground vibrational state. A two-state analysis of the vibration–rotation interaction for the v=0 and v=1 states gives an energy separation between these states (inversion splitting) of $\Delta E_{01}=4689.7095\left(10\right)\;\mathrm{MHz}$. This large-amplitude motion leads to vibration–rotation coupling of energy levels. A symmetric double-minimum inversion potential function was determined, resulting in a barrier of about 45 cm^−1^ in good agreement with that obtained by DFT quantum chemical calculations.

## 1. Introduction

Polycyclic aromatic hydrocarbon (PAH) molecules are present in Earth’s atmosphere and play a major role in its chemistry. Urban areas have been found to be major sources of atmospheric PAHs, many of which are well known as human carcinogens [1]. They are primarily formed by incomplete combustion of fossil fuels, biomass, and waste [2]. They contribute to air quality and climate change and can affect human health directly or indirectly. However, while some PAHs in the gas phase are toxic to human health, others could have biological and pharmaceutical applications.

In addition to this wealth of information, PAHs have been proposed as the primary carriers of the dominant unidentified infrared emission bands (UIBs) observed in numerous galactic and extragalactic sources [3]. The intensity of these UIBs indicates that PAHs play a relevant role in the carbon chemistry of the interstellar medium (ISM), since around 10–25% of the carbon in the Milky Way is fixed in these forms [3]. The first aromatic molecule detected in the infrared, benzene, was identified in the protoplanetary carbon-rich star CRL618 [4]. The advances in rotational spectroscopy have enabled the identification and characterization of several PAH molecules [5,6,7] and the detection of many of them in space [8,9]. Recent studies have confirmed the presence of various cyano- and ethynyl-substituted PAHs up to seven aromatic rings [10,11,12], further expanding our understanding of the formation of complex organic molecules in the interstellar medium [13,14,15,16,17,18].

**Figure 1:**
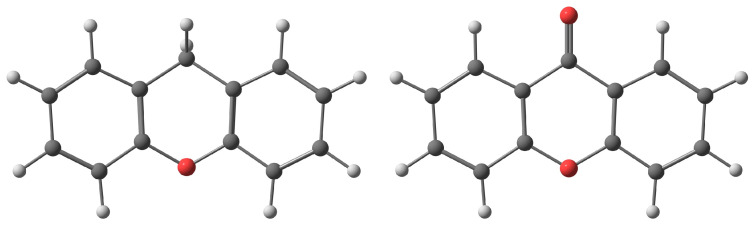
Molecular structure of xanthene (**left**) and xanthone (**right**) molecules.

Xanthenes are aromatic compounds characterized by a three-ring structure, consisting of two benzene rings connected to a central pyran ring. Over the past two decades, xanthene derivatives have garnered significant attention for their applications targeting biological systems [19,20]. Numerous interesting derivatives have been documented in the scientific literature and patent applications, highlighting various synthetic strategies and bioactivities. These compounds exhibit a variety of biological activities, including antitumor, antibacterial, anti-Alzheimer, anti-inflammatory, and anti-diabetic effects [19,20,21]. In addition to their pharmaceutical applications, xanthenes are also used in laser technology, the food industry, and the production of fluorescent materials [22]. On the one hand, xanthene (9H-xanthene, 10H-9-oxaanthracene), with a molecular formula C_13_H_10_O (see Figure 1), serves as the core structure for various aromatic compounds, including dyes such as fluorescein and rhodamine. Then, it is used in organic synthesis and material science for fluorescence applications. On the other hand, xanthone (9H-Xanten-9-ona, dibenzo-γ-pirona, see Figure 1) is a natural compound that occurs as a secondary metabolite in a wide range of terrestrial and marine sources [23,24]. Xanthone is also a versatile scaffold in organic synthesis, often employed to construct complex molecular architectures and to develop new therapeutic agents. The bioactivities of xanthones makes them valuable in research on medicinal chemistry in pharmaceutical and biological aspects [25,26,27]. Both compounds, xanthene and xanthone, are related by an oxidation reaction: xanthone has been reported as the oxidation product of xanthene in several studies using different methods [28,29,30,31]. All this information makes them interesting to be investigated in the gas phase to understand their behavior and to provide their spectroscopic parameters, for example, to detect them in space.

High-resolution rotational spectroscopy is a powerful technique employed alongside quantum chemical calculations, providing comprehensive insights into molecular structures, large-amplitude motions, hyperfine structures, and non-covalent interactions of molecules and molecular aggregates in the gas phase [32,33,34,35,36]. On top of that, it provides essential information (rotational constants) that permits the identification of the chemical species in various environments such as the ISM. The rotational spectrum of xanthene has been recently studied in the 2–8 GHz range using a chirped-pulse Fourier transform microwave (CP-FTMW) spectrometer, showing a single non-planar conformation where two splitting states were observed [37]. However, since the two states were fitted separately, no information about the energy separation between the two levels was obtained and the inversion barrier height was only estimated based on density functional theory (DFT) B3LYP calculations. Furthermore, the determination of a good energy difference and Coriolis coupling between the two states allows for a better understanding of the internal movements of the species and for accurately determining the rotational parameters necessary for future detection of xanthene in space.

In the present paper, we report a high-resolution study of xanthene and its oxidation product xanthone using a Fourier transform microwave spectroscopy technique coupled with a pulsed molecular jet-expansion, where the inversion motion in the ground state has been analyzed and modeled using a one-dimensional potential energy function. Furthermore, through our investigation of xanthene, we surprisingly observed that the presence of water in the carrier gas in high amounts leads to a decrease in the signal of the xanthene, and new signals appear instead. The new signals were assigned to the oxidation product xanthone. Interestingly, analysis of the rotational spectrum reveals that both species show different structural behaviors. In contrast to other oxidation processes, such as β-pinene to nopinone [38] and α-pinene to α-pinene oxide [39,40] where both precursor and product structures have been shown to retain very similar geometries. In this case, while xanthene has an inversion motion derived from its non-planarity, its oxidation blocks this inversion, causing xanthone to become a planar molecule with subsequent higher connectivity between the two aromatic rings.

## 2. Results and Discussion

### 2.1. Xanthene Analysis

**Figure 2:**
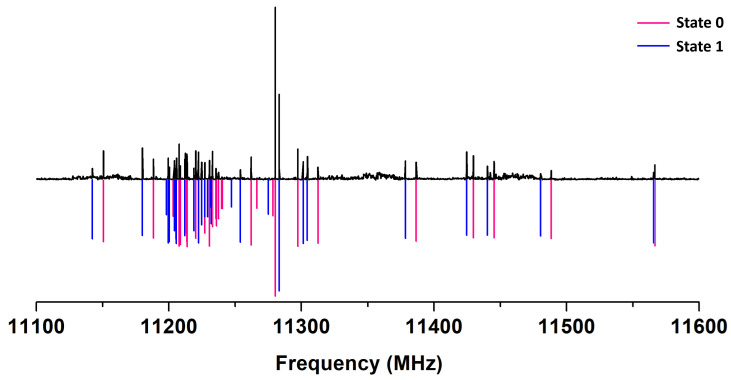
A portion of the low-resolution experimental survey in the Concerning the line intensity ratios of the two levels, a previous study of xanthene inferred a 3:1 statistical ratio between 11,100–11,600 MHz [37]. range showing the observed (upper part) and the simulated (lower part, $T_{rot}=5$ K) rotational lines of xanthene with the two sub-states. The intensity is in arbitrary units. The spectrum has been recorded at a repetition rate of 2 Hz and 35 averages per step of 250 kHz.

Xanthene is an asymmetric top with a mainly $\mu_{b}$ electric dipole moment component. Its predicted spectrum is dense with many pronounced transitions of the R- and Q-branches. As mentioned, the experimental spectrum is denser than expected due to the inversion motion of the two benzene rings, see Figure 2. The measured intra- and inter-states transitions of the two inversion sub-states (here called 0 and 1) were fitted simultaneously in a two-state coupled fit based on Pickett’s reduced axis Hamiltonian [41]. The Hamiltonian is in 2×2 block-diagonal form:

(1) $$H=\left(\begin{array}{cc} H_{0}^{\mathrm{rot}}+H_{0}^{\mathrm{CD}} & H^{\mathrm{int}}\\ H^{\mathrm{int}} & H_{1}^{\mathrm{rot}}+H_{1}^{\mathrm{CD}}+\Delta E_{01} \end{array}\right)$$

where the diagonal blocks correspond to a single-state Watson’s Hamiltonian in the S-reduction and $I^{r}$ representation ($H^{\mathrm{rot}}$) supplemented with the centrifugal distortion correction $H^{\mathrm{CD}}$ [42]. $\Delta E_{01}$ is the vibrational energy difference $E_{1}-E_{0}$ between the two sub-states. The off-diagonal terms represent the Coriolis coupling term given by $H^{\mathrm{int}}=F_{ac}\times \left(P_{a}P_{c}+P_{c}P_{a}\right)$ and consider all interactions between the two states. Many line frequencies were observed to be perturbed due to Coriolis coupling interactions. These lines are very important to determine the energy difference between the sub-levels. By fitting the perturbed lines, it was possible to determine the energy difference between the two sub-states. After obtaining precise rotation parameters, c-type interstate transitions were predicted. These transitions are essential for further enhancing the energy difference. Although xanthene exhibits only a small dipole moment along the c-axis ($|\mu_{c}|∼0.1$ D), our spectrometers are capable of detecting such low-dipole moments thanks to their exceptional sensitivity [38,40,43], allowing us to successfully detect several c-type interstate transitions, improving further the rotational parameters and especially the energy difference value $\Delta E_{01}$, supporting the possible future detection of xanthene in space.

In total, 511 rotational lines were fitted together at instrumental accuracy. The fitted molecular parameters are reported in Table 1 and compared to those predicted by different calculation methods. A very good agreement is observed between the experimental rotational parameters and the calculated methods M06-2X/6-311++G(d,p) and ωB97xD/6-311++G(d,p). An acceptable performance was also observed at the B3LYP/6-311++G(d,p) level, while the poorest performance was obtained using the ab initio method MP2/6-311++G(d,p).

**Figure 3:**
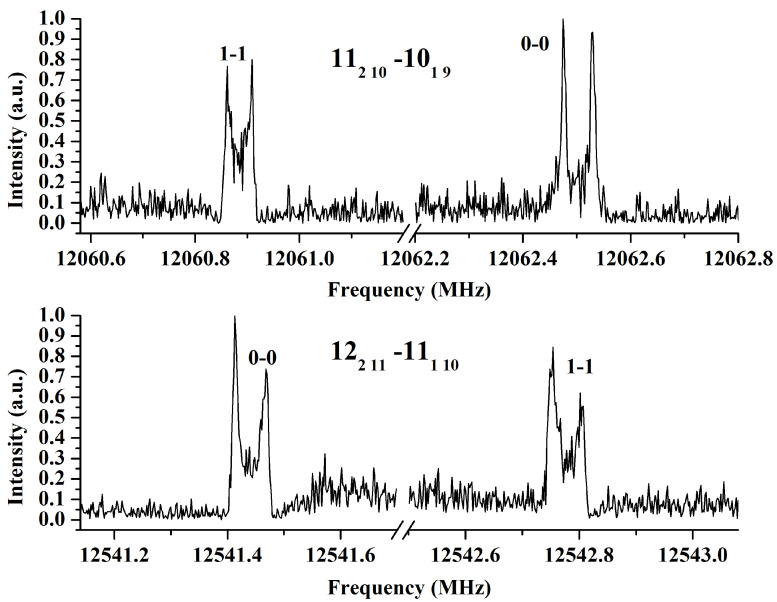
High-resolution line transitions from the two sub-states of xanthene. The intensity has been normalized and it shows a ratio of almost 0.8 between them. The lines have been recorded using the same experimental conditions (700 shots for upper lines and 1000 for lower lines). The $J_{Ka\;Kc}$ states quantum numbers are indicated.

Concerning the line intensity ratios of the two levels, a previous study of xanthene inferred a 3:1 statistical ratio between the tunneling components, leading to an inversion of the two equivalent hydrogen atoms of the –CH_2_ group [37]. As shown in Figure 3, after checking several lines recorded under the same conditions and using the same cavity mode, the observed ratio is not 3:1. The lines from the two sub-levels have relative intensities of ∼0.8. This is coherent with the small energy value separating the two sub-levels (4689.7095(10) MHz, 0.156431871(33) cm^−1^), as detailed in Section 3.2. Consequently, the large-amplitude motion in xanthene involves the rings and the splitting intensity cannot be only characterized by the CH_2_ proton tunneling as previously suggested [37].

### 2.2. Barrier to Rings Inversion

Xanthene exhibits an out-of-plane bending of its atoms in the aromatic rings, leading to a symmetrical double-potential minimum in the ground state. As shown in the figure of Table 2, the barrier height between both minima corresponds to the planar structure of xanthene. The energy of this inversion barrier has been predicted using theoretical methods. The calculated barriers using DFT methods indicate that the barrier height is relatively low (35 to 55 cm^−1^), while the same barrier was found to be 20 times higher using the ab initio MP2 method (748 cm^−1^). These results are summarized in Table 3.

Our rotational analysis including the two sub-states observed in the spectra allowed us to determine the energy difference between them ($\Delta E_{01}=4689.7095\left(10\right)$ MHz), suggesting that the barrier height to the motion is small. The ground state inversion barrier has been estimated to be about 49 cm^−1^ from a Laser-Induced Fluorescence study [44]. Using the experimentally determined value $\Delta E_{01}$, the inversion motion was modeled using a reduced quartic–quadratic potential of the following form:

(2) $$V\left(z\right)=A\left(z^{4}+Bz^{2}\right)$$

Here, *z* is a dimensionless coordinate and *B* is a negative constant for a double-minimum potential. The barrier height is located at $V_{0}=\frac{AB^{2}}{4}$ and $z_{\min}^{2}=-\frac{B}{2}$. This potential form has been described in detail by Laane [45]. The double-minimum potential of xanthene was fitted using the ANHARM software [46], with 50 harmonic wavefunctions, to the following form:

(3) $$V\left(z\right)=4.764\times \left(z^{4}-6.15z^{2}\right)$$

which results in a barrier height of 45 cm^−1^. This value closely matches the 49 cm^−1^ barrier determined by Chakraborty et al. [44] using a Gaussian-type double-minimum potential. Both energy levels and splitting values show excellent agreement with the previous study. The model predicts an energy difference of $\Delta E_{01}=4731$ MHz, aligning nicely with our experimental result of 4689.7095(10) MHz. Table 2 contains the comparison between our model and the experimental values of the bibliography.

When comparing the different computational approaches (Table 3), MP2/6-311++G(d,p) performs poorly in predicting a reliable inversion barrier for xanthene. DFT methods, however, yield very good estimates. Among them, ωB97xD/6-311++G(d,p) provides the best match to the experimental data, followed closely by the M06-2X method. The calculated angle deviation from planarity across all DFT methods is close to 20°, which agrees with the value determined using Kraitchman’s method [37]. Again, MP2 underperforms in the prediction of the structure of xanthene. Given that the MP2/6-311++G(d,p) level of theory is known to overestimate the energy of bent structures [47], additional calculations were performed using the more reliable aug-cc-pVTZ correlation-consistent basis set. The resulting barrier height is approximately 158 cm^−1^, which, although closer to the model prediction, remains 3.5 times higher than the value derived from experimental observations.

### 2.3. Xanthone Analysis

Once the transitions of xanthene were assigned, a remaining set of unassigned lines could be identified. By surveying these lines, it was observed that the remaining ones are linked to the presence of water in the carrier gas. Their intensities increase with high water content and are further accelerated by heating. Temperature variation tests (380 to 420 K) revealed that higher temperatures yield these lines faster, while the xanthene lines diminish significantly. These lines, exhibiting a b-type transition pattern, were successfully fitted and a new set of experimental parameters was derived, leading to the identification of a new species in the spectra. Additionally, the use of lower polarization power implies that this species has a much higher dipole moment than xanthene. A total of 336 transition lines were fitted using the same Watson’s Hamiltonian in the S-reduction and $I^{r}$ representation ($H^{\mathrm{rot}}$), supplemented with the centrifugal distortion Hamiltonian ($H^{\mathrm{CD}}$) [42]. The smaller rotational constants suggest that the observed species is heavier than xanthene, as confirmed in Table 4.

**Figure 4:**
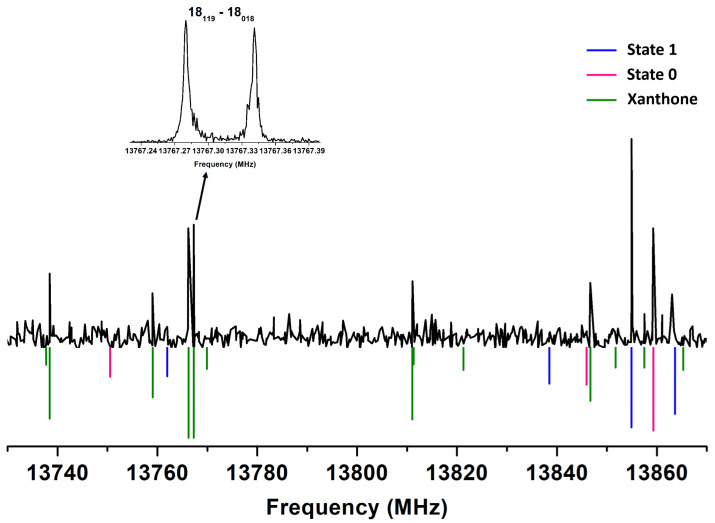
Low-resolution experimental survey in the 13,730–13,870 MHz range in the presence of a small amount of water in the carrier gas (upper part). The lower part consists of a simulation of the rotational spectra (Trot = 5 K) of the two sub-states of xanthene (red and blue) and its oxidation product xanthone (green). Intensity is in arbitrary units. The spectrum has been recorded at a repetition rate of 2 Hz with a step of 250 kHz and 35 averages. The inset shows a high-resolution transition of xanthone (200 averages) with related $J_{Ka\;Kc}$ quantum numbers.

The new species is only observed with water present. Figure 4 shows the emergence of these new lines in the presence of water. One might think that the lines belong to xanthene–water complexes, but this thought was discarded since these lines are not attributable to xanthene–water complexes, according to previous studies [37]. Subsequently, we suspected that water might trigger a reaction in our instrument. In the literature, earlier studies indicate that xanthene oxidation yields xanthone [28,29,30]. The comparison of molecular parameters from theoretical calculations confirmed xanthone’s identification. First, the experimental rotational constants match those from theoretical calculations with less than 1% error. Centrifugal distortion constants also align closely. Second, the observation of only b-type transitions, consistent with the microwave power used, also supports the identification of xanthone. Third, xanthone’s planar and rigid structure, lacking inversion motion, matches predictions from quantum chemistry calculations. Consequently, xanthone is obviously present in our experiments via the degradation of xanthene. Thus, the oxidation reaction previously observed in the literature with high yield [28,29,30] appears to have occurred in our experiments without catalysis, similar to esterification reactions observed in the gas phase [48].

### 2.4. Structural Investigation

The oxidation of xanthene to xanthone produces significant structural changes. As confirmed by our study, the non-planar xanthene presents a large amplitude motion involving the two aromatic rings, contrary to the proton tunneling suggested previously [37]. This movement is blocked after the oxidation, resulting in a planar structure of xanthone. On top of that, this oxidation of the benzylic carbon leads to a structure that is over-stabilized by the presence of further H-bond interactions.

The rigidity of xanthene’s structure is clearly deduced from centrifugal distortion values, with small quartic distortion constants. In contrast, xanthene shows higher $D_{JK}$ and $D_{K}$ values due to its inversion motion. This highlights how the methylene group (CH_2_) restricts full planarization of the xanthene molecule, compared to the ketone group (C=O) formed after the oxidation.

**Figure 5:**
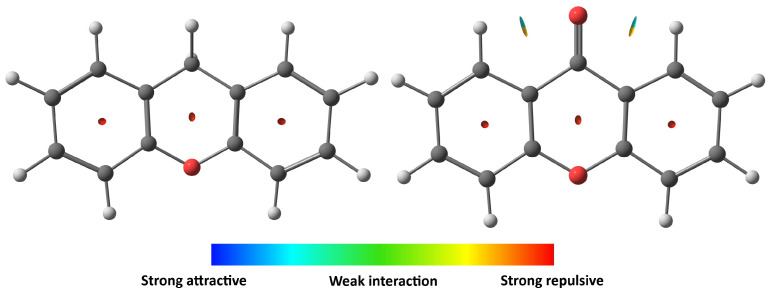
NCI plot highlighting the intra-molecular hydrogen bonding in the xanthone molecule (**right**) compared to the xanthene molecule (**left**). The blue-green iso-surfaces indicate a relatively medium hydrogen bond interaction between the hydrogens and the oxygen of xanthone.

To explore their behavior, non-covalent intermolecular interactions (NCIs) were analyzed using NCI plot analysis of the electron density and its derivative [49]. Multiwfn software [50] was used based on the M06-2X/6-311++G(d,p) level of theory outputs, for both molecules. The analysis indicates relatively medium hydrogen bonding between the ketone oxygen of xanthone and adjacent benzene hydrogen, enforcing planarity, unlike xanthene, which lacks such intramolecular bonding as shown in Figure 5.

**Figure 6:**
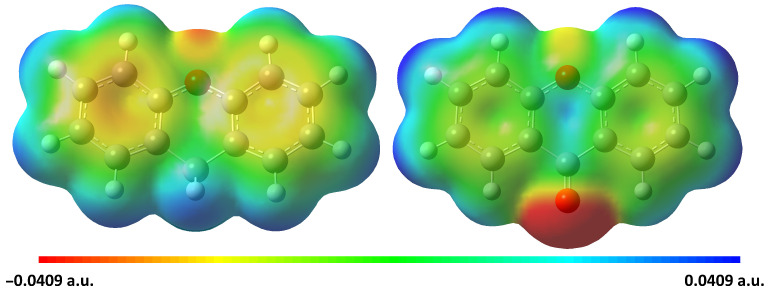
Molecular electrostatic potential (MEP) maps of xanthene (**left**) and xanthone (**right**) computed at M06-2X/6-311++G(d,p) showing the electron density isosurface. The different color gradient represents the electrostatic potential, where positive potential is shown in blue and negative potential in red. The electron density values range from −0.0409 to 0.0409 a.u. for both molecules.

Molecular electrostatic potential (MEP) is a powerful tool for visualizing the spatial distribution of charge within a molecule. It effectively highlights regions of variable electrostatic potential, making it useful for identifying the most likely sites of electrophilic and/or nucleophilic reactivity [51]. Using DFT calculations at the M06-2X/6-311++G(d,p) level, MEP plots (see Figure 6) show regions of high negative potential (red) and positive potential (blue). The electrostatic potential increases in the following order: red < orange < yellow < green < blue. The red color describes areas with the greatest negative electrostatic potential, which is the preferred site for electrophilic attack. In contrast, blue areas are those that have the lowest density and the highest positive electrostatic potential, which is the preferred site for nucleophilic attack. In xanthene, the negative potential is located near the oxygen atom, while the positive potential is centered around methylene hydrogen atoms, likely the reactive centers for a nucleophilic attack. The positively charged sites (mainly the CH_2_) should be involved in electrophilic attack during oxidation when water is present, providing some hints about the mechanism of the oxidation process. This interpretation is fully consistent with the observation of the oxidation product xanthone, where the two hydrogen atoms of the methylene group are replaced by the ketone group. Notably in xanthone, the ketone oxygen becomes more negatively charged than the ring oxygen, illustrating a shift in charge distribution upon oxidation and contributing to its planarity by means of medium hydrogen bonding with adjacent hydrogen atoms.

## 3. Materials and Methods

### 3.1. Theoretical Calculations

Theoretical calculations are necessary to predict molecular properties and to assist in the analysis of rotational spectra. The GAUSSIAN16 software package [52] was used to apply DFT and ab initio methods to examine the molecular parameters of xanthene and its oxidation product xanthone. The optimizations were carried out using the ab initio Møller–Plesset second-order perturbation theory (MP2) [53] and the DFT methods B3LYP [54,55], ωB97xD [56], and M06-2X [57] with the Pople split-valence triple-zeta basis set augmented with diffuse and polarization functions on all atoms (the 6-311++G(d,p) basis set) [58]. Additional calculations were performed using MP2 with the Dunning’s correlation-consistent triple-zeta aug-cc-pVTZ basis set with added polarization functions [59]. In particular, the barrier heights of the inversion motion were investigated using all these methods to be compared to the model value obtained from experimental data.

### 3.2. Experimental

The pure rotation spectra of xanthene and xanthone were recorded in the 2–20 GHz frequency range using two Fabry–Perot supersonic jet Fourier transform microwave (FP-FTMW) spectrometers (optimal working ranges: 2–12 GHz and 10–20 GHz). The experimental setups are described in detail elsewhere [60,61]. Xanthene (99%) purchased from Sigma Aldrich was used without further purification. It was placed inside the reservoir of a nozzle where it was heated up to about 400 K and mixed with argon as carrier gas at a backing pressure of about 0.2 MPa. The mixture was then introduced into the cavity along the optical axis, through a 1 mm diameter pinhole using a pulsed nozzle at a repetition rate of 2 Hz. The rotational temperature of the molecules in the supersonic jet was estimated to a few Kelvin.

Microwave power pulses of 2 μs were used to polarize the molecules. Free-Induction Decay signals were detected and digitized at a repetition rate of 120 MHz on a 14-bit resolution electronic card. After a fast Fourier transformation of the time-domain signals, lines were observed as Doppler doublets. The central frequency of each line was determined by averaging the frequencies of the two Doppler components. The spectral resolution used in these experiments was higher than 4 kHz.

Xanthone lines were detected during the spectral surveys of xanthene. Their appearance correlates strongly with the presence of water in the carrier gas: as the xanthone signal increases, the xanthene lines diminish. To enhance the production of xanthone in our experiments, we added water to the carrier gas to increase its signal by promoting the degradation of xanthene. This strategy was based on our observations that xanthone lines emerge only in the presence of water, typically after several hours, and are further enhanced by heating. In contrast, under identical conditions but without water—even with prolonged heating—no xanthone signals were detected. These results indicate that water plays a key role in the oxidation of xanthene to xanthone under our experimental conditions.

## 4. Conclusions

In this work, we have provided a comprehensive spectroscopic characterization of xanthene and its oxidation product, xanthone, in the gas phase using high-resolution Fourier transform microwave spectroscopy combined with quantum chemistry calculations. The non-planar equilibrium structure of xanthene, due to the puckering of the central pyran ring, gives rise to a large-amplitude inversion motion. This motion leads to a tunneling splitting in the ground state, resulting in two distinct sub-levels (*v* = 0 and *v* = 1), which were successfully fitted using a reduced axis Hamiltonian. The experimentally determined inversion splitting of $\Delta E_{01}=4689.7095\left(10\right)\;\mathrm{MHz}$ enabled the construction of a double-minimum potential, yielding a barrier height of approximately 45 cm^−1^, consistent with the best-performing DFT methods (ωB97xD and M06-2X).

Interestingly, under experimental conditions involving water in the argon carrier gas, we observed the in situ oxidation of xanthene to xanthone. The identification of xanthone was unambiguous, based on its b-type rotational spectrum, showing a significantly higher dipole moment, and rotational constants that matched theoretical predictions within a deviation of less than 1%. The xanthene to xanthone reaction involves oxidation at the benzylic carbon, producing a conjugated ketone structure that induces planarity through intramolecular hydrogen bonding, as visualized by non-covalent interaction plots. This structural rigidity is reflected in the notably smaller centrifugal distortion constants for xanthone compared to xanthene. Molecular electrostatic potential analysis further supports this reaction pathway: the positive potential localized at the methylene hydrogen atoms in xanthene marks them as reactive centers, facilitating their substitution by the ketone group. In addition, our results highlight the inadequacy of MP2 for modeling the inversion barrier in the present case, with DFT functionals providing superior agreement with experimental results.

This study refines the spectroscopic constants necessary for future detection of these compounds in interstellar or atmospheric environments. It also reveals insights into the spontaneous oxidation pathway of xanthene in the presence of water.

## Figures and Tables

**Table 1:** Ground-state experimental rotational parameters of xanthene compared to the equilibrium parameters obtained using different computational chemistry methods.

Par. ^a^	0	1	ωB97xD	B3LYP	M06-2X	MP2
A (MHz)	2032.111253(62)	2031.038848(69)	2048.0	2056.6	2042.0	1904.9
B (MHz)	465.950920(23)	466.056198(24)	468.2	462.4	467.9	477.6
C (MHz)	385.125452(20)	385.251448(21)	387.3	381.9	387.1	399.4
$D_{J}$ (kHz)	0.014016(30)	0.012734(33)	0.0165	0.0098	0.0129	0.0131
$D_{JK}$ (kHz)	−0.23104(17)	−0.20487(21)	−0.281	−0.1527	−0.2110	−0.177
$D_{K}$ (kHz)	1.2645(17)	1.1285(16)	1.502	0.8841	1.1587	0.882
$d_{1}$ (kHz)	−0.000560(15)	−0.000469(17)	0.0008	0.0003	0.0006	0.0004
$d_{2}$ (kHz)	0.0001420(66)	0.0001351(70)	−0.0001	−0.0001	−0.0001	−0.0001
$\Delta E_{01}$ (MHz)	4689.7095(10)		-	-	-	-
$F_{ac}$ (MHz)	0.360263(32)		-	-	-	-
$|\mu_{a}|$ (D)	-		0.0	0.0	0.0	0.0
$|\mu_{b}|$ (D)	-		1.1	1.1	1.1	1.1
$|\mu_{c}|$ (D)	-		0.1	0.1	0.1	0.2
N ^b^	511		-	-	-	-
σ ^c^ (kHz)	2.8		-	-	-	-

^a^ A, B, and C are the rotational constants given in MHz. $D_{J}$, $D_{JK}$, $D_{K}$, $d_{1}$, and $d_{2}$ are the quartic centrifugal distortion constants in kHz. $\Delta E_{01}$ is the vibrational energy difference between the two sub-states in MHz. $F_{ac}$: Coriolis coupling in MHz. $\mu_{a}$, $\mu_{b}$, and $\mu_{c}$ are the calculated dipole moment components in Debye. The standard error is given in parentheses in units of the last digits. ^b^ Number of fitted lines. ^c^ σ is the RMS deviation of the fit in kHz.

**Table 2:** Inversion motion of xanthene. Comparison between the experimental and modeled energy levels from this work.

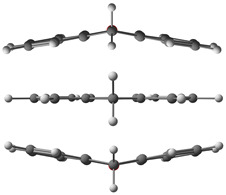		$\Delta E_{0v}$ (cm^−1^) ^c^	$\Delta E_{0v}$ (cm^−1^) ^d^
	*v*	This work model	Experimental [44]
	1	0.158	0
	2	24.7	26
	3	29.4	30
	4	45.3	46
	5	60.2	60
	6	78.0	78
	7	97.6	97
	8	119	119
$\Delta E_{01}$ = 0.15643190(3) ^a,b^	Barrier	45	49

^a^ The figure shows the two equivalent minima of the inversion motion (upper and lower) and the structure at the barrier height (middle). ^b^ $\Delta E_{01}$ corresponds to the energy difference between the two sub-states obtained from our rotational analysis fit. ^c^ $\Delta E_{0v}$ is the energy difference between the ground state and the *v* vibrational level obtained from our model (see Section 2.2). ^d^ Experimental data of $\Delta E_{0v}$ from the literature [44]. The energy value for *v* = 1 was not experimentally fully determined.

**Table 3:** Calculated barrier of inversion motion of xanthene using different theoretical methods. All calculations used 6-311++G(d,p) basis set.

Method	Barrier Height (cm^−1^)	Angle to Planarity (°)
B3LYP	34	162.1
M06-2X	54.7	158.5
ωB97xD	44.4	158.7
MP2	748	144.8

**Table 4:** Ground-state experimental rotational parameters of xanthone compared to equilibrium parameters obtained using different computational chemistry methods.

Par.	Exp. ^a^	ωB97xD	B3LYP	M06-2X	MP2
A (MHz)	1461.290022(36)	1472.3	1463.0	1471.6	1451.5
B (MHz)	465.138292(19)	467.4	464.1	466.8	463.4
C (MHz)	352.988498(14)	354.7	352.3	354.4	351.3
$D_{J}$ (kHz)	0.001721(20)	0.0017	0.0017	0.0017	0.0017
$D_{JK}$ (kHz)	0.00700(12)	0.0069	0.0069	0.0067	0.0070
$D_{K}$ (kHz)	0.03076(48)	0.0289	0.0291	0.0291	0.0287
$d_{1}$ (kHz)	−0.000493(10)	−0.0005	−0.0005	−0.0005	−0.0005
$d_{2}$ (kHz)	−0.0000884(48)	−0.0001	−0.0001	−0.0001	−0.0001
$|\mu_{a}|$ (D)	-	0.0	0.0	0.0	0.0
$|\mu_{b}|$ (D)	-	3.0	3.1	2.8	2.8
$|\mu_{c}|$ (D)	-	0.0	0.0	0.0	0.0
N ^b^	336	-	-	-	-
σ ^c^ (kHz)	2.3	-	-	-	-

^a^ A, B, and C are the rotational constants given in MHz. $D_{J}$, $D_{JK}$, $D_{K}$, $d_{1}$, and $d_{2}$ are the quartic centrifugal distortion constants in kHz. $\mu_{a}$, $\mu_{b}$, and $\mu_{c}$ are the calculated dipole moment components in Debye. The standard error is given in parentheses in units of the last digits. ^b^ Number of fitted lines. ^c^ σ is the RMS deviation of the fit in kHz.

## Data Availability

The data presented in this study is available in the Supplementary Materials.

## Supplementary Materials

The following supporting information can be downloaded at https://www.mdpi.com/article/10.3390/molecules30132801/s1: Table S1: Deviation of the molecular parameters of xanthene. Table S2: Deviation of the molecular parameters of xanthone. Table S3: Measured rotational transitions of the two sub-states of xanthene. Table S4: Measured rotational transitions of xanthone. Table S5: Principal axis Cartesian coordinates of xanthene from M06-2X/6-311++G(d,p) calculation. Table S6: Principal axis Cartesian coordinates of xanthone from M06-2X/6-311++G(d,p) calculation.

## Abbreviations

The following abbreviations are used in this manuscript:

PAH	polycyclic aromatic hydrocarbons
UIB	unidentified infrared emission bands
ISM	interstellar medium
CP-FTMW	chirped pulsed Fourier transform microwave
FP-FTMW	Fabry–Perot Fourier transform microwave
DFT	density functional theory
RMS	root mean square
NCI	non-covalent interaction
MEP	molecular electrostatic potential
