# Supplementary material for: Inversion Motion of Xanthene and Detection of Its Oxidation Product Xanthone from Gas-Phase Rotational Spectroscopy
Source: Molecules. 2025 Jun 29;30(13):2801. doi: 10.3390/molecules30132801 (PMC12251198; doi:10.3390/molecules30132801)
Supplement: Supplementary file 1 [file molecules-30-02801-s001.zip › molecules-3700681-supplementary.pdf]

Supplementary materials

Inversion motion of Xanthene and detection of its oxidation product Xanthone from gas phase rotational spectroscopy

Celina Bermúdez[1], Manuel Goubet[2], and Elias M. Neeman[2]

[1]Departamento de Química Física y Química Inorgánica, Facultad de Ciencias — I.U. CINQUIMA, Paseo de Belén 7, 47011 Valladolid, Spain
[2]Univ. Lille, CNRS, UMR8523 - PhLAM - Physique des Lasers Atomes et Molécules, F-59000 Lille, France

Table S1: **Deviation of the molecular parameters of xanthene.**

**Constants**	**ωB97xD**	**B3LYP**	**M06-2X**	**MP2**
A	-0.8	-1.2	-0.5	6.3
B	-0.5	0.8	-0.4	-2.5
C	-0.6	0.8	-0.5	-3.7
D_J	-17.8	30.4	8.0	6.6
D_{JK}	-21.8	33.9	8.7	23.3
D_K	-18.8	30.1	8.3	30.2
d_1	-57.2	49.6	-0.5	29.3
d_2	-11.0	23.6	5.9	18.0

Calculated deviation in % using $\left(\frac{Exp-Theo}{Exp}\right) \times 100$*, based on the experimental data obtained for the 0 sub-state. The same number of digits has been used to calculate the error on the centrifugal distortion constants.*

Table S2: **Deviation of the molecular parameters of xanthone.**

**Constants**	**ωB97xD**	**B3LYP**	**M06-2X**	**MP2**
A	-0.8	-0.1	-0.7	0.7
B	-0.5	0.2	-0.4	0.4
C	-0.5	0.2	-0.4	0.5
D_J	0.3	-0.3	0.4	0.6
D_{JK}	1.7	1.0	4.1	0.2
D_K	6.6	6.0	6.1	7.1
d_1	3.1	2.9	2.6	3.9
d_2	-4.8	-4.8	-4.8	-7.1

Calculated deviation in % using $\left(\frac{Exp-Theo}{Exp}\right) \times 100$*. The same number of digits has been used to calculate the error on the centrifugal distortion constants.*

Table S3: **Measured rotational transitions of xanthone.**

J'	K'_a	K'_c	V'	J''	K''_a	K''_c	V''	obs[a]	obs. - calc[b]
7	0	7	0	6	1	6	0	4876.9556	-0.001
8	0	8	0	7	1	7	0	5820.4009	0.000
9	0	9	0	8	1	8	0	6743.2355	0.002
10	0	10	0	9	1	9	0	7642.3991	0.000
11	0	11	0	10	1	10	0	8517.6818	0.000

12	0	12	0	11	1	11	0	9370.9227	-0.001
13	0	13	0	12	1	12	0	10205.1032	0.001
14	0	14	0	13	1	13	0	11023.5773	0.002
15	0	15	0	14	1	14	0	11829.5726	-0.001
16	0	16	0	15	1	15	0	12625.9382	0.000
17	0	17	0	16	1	16	0	13415.0248	-0.001
18	0	18	0	17	1	17	0	14198.7145	0.001
19	0	19	0	18	1	18	0	14978.4518	0.001
20	0	20	0	19	1	19	0	15755.3327	-0.001
21	0	21	0	20	1	20	0	16530.1729	0.000
22	0	22	0	21	1	21	0	17303.5612	-0.001
23	0	23	0	22	1	22	0	18075.9283	-0.003
13	1	12	0	12	2	11	0	9096.1737	-0.002
14	1	13	0	13	2	12	0	10213.2463	-0.001
15	1	14	0	14	2	13	0	11297.3164	0.000
16	1	15	0	15	2	14	0	12342.9133	-0.001
17	1	16	0	16	2	15	0	13347.6570	0.000
18	1	17	0	17	2	16	0	14312.1325	-0.002
19	1	18	0	18	2	17	0	15239.2854	0.000
20	1	19	0	19	2	18	0	16133.5277	0.000
21	1	20	0	20	2	19	0	16999.9099	-0.001
22	1	21	0	21	2	20	0	17843.4707	-0.001
23	1	22	0	22	2	21	0	18668.8263	0.001
16	2	14	0	15	3	13	0	9055.5389	-0.001
19	2	17	0	18	3	16	0	13055.2745	-0.005
20	2	18	0	19	3	17	0	14347.6173	-0.003
21	2	19	0	20	3	18	0	15601.4901	0.002
22	2	20	0	21	3	19	0	16808.5235	0.002
23	2	21	0	22	3	20	0	17963.1800	0.000
3	2	1	0	2	1	2	0	7509.3879	-0.001
5	2	3	0	4	1	4	0	9582.9579	-0.001
6	2	4	0	5	1	5	0	10737.1625	0.004
7	2	5	0	6	1	6	0	11987.4456	0.004
8	2	6	0	7	1	7	0	13345.7382	-0.004
9	2	7	0	8	1	8	0	14821.0192	0.004
10	2	8	0	9	1	9	0	16418.3701	0.003
3	3	0	0	2	2	1	0	10587.9709	-0.001
4	3	1	0	3	2	2	0	11445.3698	-0.002
5	3	2	0	4	2	3	0	12310.0701	0.000
6	3	3	0	5	2	4	0	13186.7326	-0.001
7	3	4	0	6	2	5	0	14081.4545	-0.002

8	3	5	0	7	2	6	0	15001.9503	0.000
9	3	6	0	8	2	7	0	15957.7063	0.000
10	3	7	0	9	2	8	0	16960.0727	0.000
11	3	8	0	10	2	9	0	18022.1537	0.001
4	4	0	0	3	3	1	0	14650.6445	0.001
5	4	1	0	4	3	2	0	15502.6335	-0.001
6	4	2	0	5	3	3	0	16354.3854	0.000
7	4	3	0	6	3	4	0	17205.9423	0.000
8	4	4	0	7	3	5	0	18057.7128	0.000
5	5	0	0	4	4	1	0	18714.6537	0.001
5	5	1	0	4	4	0	0	18714.6537	0.002
5	1	5	0	4	0	4	0	5280.8363	0.000
6	1	6	0	5	0	5	0	5926.6863	0.002
7	1	7	0	6	0	6	0	6561.3854	-0.002
8	1	8	0	7	0	7	0	7194.7854	-0.017
9	1	9	0	8	0	8	0	7835.6209	-0.002
10	1	10	0	9	0	9	0	8490.2073	0.000
11	1	11	0	10	0	10	0	9162.0381	-0.001
12	1	12	0	11	0	11	0	9851.9334	0.002
13	1	13	0	12	0	12	0	10558.7579	0.000
14	1	14	0	13	0	13	0	11280.2942	0.000
15	1	15	0	14	0	14	0	12013.9300	0.000
16	1	16	0	15	0	15	0	12757.1259	0.000
17	1	17	0	16	0	16	0	13507.6623	0.010
18	1	18	0	17	0	17	0	14263.6737	0.001
19	1	19	0	18	0	18	0	15023.7418	0.002
20	1	20	0	19	0	19	0	15786.7455	0.000
21	1	21	0	20	0	20	0	16551.8587	-0.001
22	1	22	0	21	0	21	0	17318.4733	0.000
23	1	23	0	22	0	22	0	18086.1427	-0.002
2	2	1	0	1	1	0	0	6481.4480	0.002
3	2	2	0	2	1	1	0	7251.7082	0.004
5	2	4	0	4	1	3	0	8670.1094	-0.009
6	2	5	0	5	1	4	0	9319.5685	0.000
7	2	6	0	6	1	5	0	9931.0099	0.000
8	2	7	0	7	1	6	0	10506.8173	0.000
9	2	8	0	8	1	7	0	11050.4528	-0.001
10	2	9	0	9	1	8	0	11566.7346	0.001
11	2	10	0	10	1	9	0	12062.4937	-0.008
12	2	11	0	11	1	10	0	12541.4406	-0.001
13	2	12	0	12	1	11	0	13019.1908	0.004

14	2	13	0	13	1	12	0	13501.6476	0.004
15	2	14	0	14	1	13	0	13998.8799	0.001
16	2	15	0	15	1	14	0	14519.1782	0.001
17	2	16	0	16	1	15	0	15068.4457	0.001
18	2	17	0	17	1	16	0	15649.7000	0.000
19	2	18	0	18	1	17	0	16263.1335	-0.001
20	2	19	0	19	1	18	0	16906.6943	-0.001
21	2	20	0	20	1	19	0	17576.9009	-0.001
22	2	21	0	21	1	20	0	18269.6416	-0.001
3	3	1	0	2	2	0	0	10584.8518	-0.001
4	3	2	0	3	2	1	0	11429.6594	-0.002
5	3	3	0	4	2	2	0	12262.6410	0.001
6	3	4	0	5	2	3	0	13075.6369	0.000
7	3	5	0	6	2	4	0	13859.2823	0.000
8	3	6	0	7	2	5	0	14604.1468	-0.001
9	3	7	0	8	2	6	0	15302.1502	0.000
10	3	8	0	9	2	7	0	15947.7727	0.001
11	3	9	0	10	2	8	0	16538.6727	0.001
12	3	10	0	11	2	9	0	17075.5718	0.001
13	3	11	0	12	2	10	0	17561.6746	0.005
14	3	12	0	13	2	11	0	18001.9793	0.002
15	3	13	0	14	2	12	0	18402.9355	0.002
4	4	1	0	3	3	0	0	14650.5691	-0.001
5	4	2	0	4	3	1	0	15502.1187	-0.001
6	4	3	0	5	3	2	0	16352.3146	0.000
7	4	4	0	6	3	3	0	17199.7023	-0.001
8	4	5	0	7	3	4	0	18042.0673	0.001
9	4	6	0	8	3	5	0	18876.1844	-0.002
8	0	8	1	7	1	7	1	5822.9118	0.001
9	0	9	1	8	1	8	1	6745.7814	-0.001
10	0	10	1	9	1	9	1	7644.8915	0.000
11	0	11	1	10	1	10	1	8519.4184	-0.002
12	0	12	1	11	1	11	1	9375.2778	0.000
13	0	13	1	12	1	12	1	10208.8483	0.000
14	0	14	1	13	1	13	1	11027.3399	0.000
15	0	15	1	14	1	14	1	11833.4648	0.001
16	0	16	1	15	1	15	1	12630.0000	0.000
17	0	17	1	16	1	16	1	13419.2844	-0.001
18	0	18	1	17	1	17	1	14203.1897	0.002
19	0	19	1	18	1	18	1	14983.1526	0.000
20	0	20	1	19	1	19	1	15760.2718	0.000

21	0	21	1	20	1	20	1	16535.3536	0.000
22	0	22	1	21	1	21	1	17308.9907	-0.001
23	0	23	1	22	1	22	1	18081.6115	-0.001
5	1	5	1	4	0	4	1	5280.9865	-0.003
12	1	11	1	11	2	10	1	7958.9209	-0.001
13	1	12	1	12	2	11	1	9100.9962	-0.001
14	1	13	1	13	2	12	1	10217.8948	-0.002
15	1	14	1	14	2	13	1	11301.4049	-0.002
16	1	15	1	15	2	14	1	12343.0782	-0.004
17	1	16	1	16	2	15	1	13355.3064	-0.003
18	1	17	1	17	2	16	1	14318.3247	-0.001
19	1	18	1	18	2	17	1	15245.1066	0.003
20	1	19	1	19	2	18	1	16139.2037	0.000
21	1	20	1	20	2	19	1	17005.5531	-0.001
22	1	21	1	21	2	20	1	17849.1528	0.001
17	2	15	1	16	3	14	1	10405.2169	-0.003
19	2	17	1	18	3	16	1	13062.9584	-0.002
20	2	18	1	19	3	17	1	14354.5858	-0.001
22	2	20	1	21	3	19	1	16828.8991	-0.003
23	2	21	1	22	3	20	1	17973.8774	0.014
2	2	0	1	1	1	1	1	6562.2091	0.000
5	2	3	1	4	1	4	1	9580.4653	0.001
6	2	4	1	5	1	5	1	10734.8576	0.003
7	2	5	1	6	1	6	1	11985.3235	0.006
8	2	6	1	7	1	7	1	13343.7856	0.006
9	2	7	1	8	1	8	1	14819.1809	0.008
10	2	8	1	9	1	9	1	16416.5375	0.001
3	3	0	1	2	2	1	1	10582.7336	0.000
4	3	1	1	3	2	2	1	11440.3673	0.000
5	3	2	1	4	2	3	1	12305.3055	-0.001
6	3	3	1	5	2	4	1	13182.2171	-0.001
7	3	4	1	6	2	5	1	14077.2018	0.000
8	3	5	1	7	2	6	1	14997.9691	-0.001
9	3	6	1	8	2	7	1	15954.0127	-0.001
10	3	7	1	9	2	8	1	16956.6737	-0.004
11	3	8	1	10	2	9	1	18019.0631	0.000
5	4	1	1	4	3	2	1	15495.4918	0.000
6	4	2	1	5	3	3	1	16347.4727	-0.001
7	4	3	1	6	3	4	1	17199.2618	0.000
8	4	4	1	7	3	5	1	18051.2654	0.000
6	1	6	1	5	0	5	1	5927.1650	-0.001

7	1	7	1	6	0	6	1	6562.2338	-0.001
9	1	9	1	8	0	8	1	7837.4210	0.000
10	1	10	1	9	0	9	1	8493.2560	0.000
11	1	11	1	10	0	10	1	9162.9817	0.000
12	1	12	1	11	0	11	1	9854.0036	0.000
13	1	13	1	12	0	12	1	10561.3363	0.000
14	1	14	1	13	0	13	1	11283.2736	0.000
15	1	15	1	14	0	14	1	12017.2629	-0.001
16	1	16	1	15	0	15	1	12760.7857	-0.001
17	1	17	1	16	0	16	1	13511.6232	0.001
18	1	18	1	17	0	17	1	14267.9378	-0.001
19	1	19	1	18	0	18	1	15028.2909	-0.001
20	1	20	1	19	0	19	1	15791.5755	0.000
21	1	21	1	20	0	20	1	16556.9637	0.000
22	1	22	1	21	0	21	1	17323.8472	-0.001
23	1	23	1	22	0	22	1	18091.7878	0.000
4	2	3	1	3	1	2	1	7978.5387	0.002
5	2	4	1	4	1	3	1	8667.7860	0.002
6	2	5	1	5	1	4	1	9317.4945	0.004
7	2	6	1	6	1	5	1	9929.1876	0.001
8	2	7	1	7	1	6	1	10505.2473	-0.001
9	2	8	1	8	1	7	1	11049.1287	-0.001
10	2	9	1	9	1	8	1	11565.5910	0.000
11	2	10	1	10	1	9	1	12060.8854	0.002
12	2	11	1	11	1	10	1	12542.7807	0.002
13	2	12	1	12	1	11	1	13020.3753	-0.001
14	2	13	1	13	1	12	1	13503.6600	-0.002
15	2	14	1	14	1	13	1	14005.1945	0.003
16	2	15	1	15	1	14	1	14518.4302	-0.001
17	2	16	1	16	1	15	1	15069.6328	0.004
18	2	17	1	17	1	16	1	15651.7627	0.000
19	2	18	1	18	1	17	1	16265.8689	0.001
20	2	19	1	19	1	18	1	16910.0067	0.000
22	2	21	1	21	1	20	1	18273.9339	0.000
3	3	1	1	2	2	0	1	10579.6119	-0.001
4	3	2	1	3	2	1	1	11424.6482	-0.001
5	3	3	1	4	2	2	1	12257.8527	0.000
6	3	4	1	5	2	3	1	13071.0682	-0.001
7	3	5	1	6	2	4	1	13854.9259	0.000
8	3	6	1	7	2	5	1	14599.9955	0.000
9	3	7	1	8	2	6	1	15298.1918	-0.001

10	3	8	1	9	2	7	1	15944.0018	0.000
11	3	9	1	10	2	8	1	16535.0848	0.001
12	3	10	1	11	2	9	1	17072.1554	0.000
13	3	11	1	12	2	10	1	17558.4041	0.000
14	3	12	1	13	2	11	1	17998.7918	-0.001
15	3	13	1	14	2	12	1	18399.5500	0.000
16	3	14	1	15	2	13	1	18767.9185	-0.001
4	4	1	1	3	3	0	1	14643.1980	0.000
5	4	2	1	4	3	1	1	15494.9755	-0.001
6	4	3	1	5	3	2	1	16345.4000	0.000
7	4	4	1	6	3	3	1	17193.0156	0.000
8	4	5	1	7	3	4	1	18035.6019	-0.001
9	4	6	1	8	3	5	1	18869.9427	0.000
5	5	1	1	4	4	0	1	18705.1554	0.000
14	1	13	0	14	0	14	0	7827.1822	0.001
17	1	16	0	17	0	17	0	10372.5546	0.000
5	3	2	0	5	2	3	0	7956.3546	0.000
6	3	3	0	6	2	4	0	7886.7355	0.000
7	3	4	0	7	2	5	0	7782.4289	0.000
8	3	5	0	8	2	6	0	7639.5946	0.000
9	3	6	0	9	2	7	0	7458.8127	0.001
10	3	7	0	10	2	8	0	7245.8956	0.000
11	3	8	0	11	2	9	0	7011.8017	0.001
5	4	1	0	5	3	2	0	11235.4873	0.000
6	4	2	0	6	3	3	0	11227.0863	-0.002
7	4	3	0	7	3	4	0	11212.4333	-0.001
8	4	4	0	8	3	5	0	11188.3537	0.000
9	4	5	0	9	3	6	0	11150.6723	0.002
10	4	6	0	10	3	7	0	11094.1545	0.000
11	4	7	0	11	3	8	0	11012.6718	0.000
12	4	8	0	12	3	9	0	10899.6301	0.003
13	4	9	0	13	3	10	0	10748.8127	0.002
14	4	10	0	14	3	11	0	10555.6337	0.003
15	4	11	0	15	3	12	0	10318.5613	0.001
16	4	12	0	16	3	13	0	10040.4382	0.001
17	4	13	0	17	3	14	0	9729.2386	0.001
6	5	1	0	6	4	2	0	14449.7055	-0.001
7	5	2	0	7	4	3	0	14445.2842	0.001
8	5	3	0	8	4	4	0	14438.5541	0.000
9	5	4	0	9	4	5	0	14428.7171	-0.001
10	5	5	0	10	4	6	0	14414.7413	0.000

11	5	6	0	11	4	7	0	14395.2772	-0.001
12	5	7	0	12	4	8	0	14368.5677	0.001
13	5	8	0	13	4	9	0	14332.3009	0.000
14	5	9	0	14	4	10	0	14283.5032	0.001
15	5	10	0	15	4	11	0	14218.3918	0.002
16	5	11	0	16	4	12	0	14132.3097	0.001
17	5	12	0	17	4	13	0	14019.7514	-0.014
18	5	13	0	18	4	14	0	13874.6613	0.001
19	5	14	0	19	4	15	0	13690.8073	0.001
20	5	15	0	20	4	16	0	13462.7972	0.000
21	5	16	0	21	4	17	0	13187.2091	0.001
6	6	0	0	6	5	1	0	17664.4311	0.000
6	6	1	0	6	5	2	0	17664.4311	0.000
8	6	2	0	8	5	3	0	17658.8089	0.002
8	6	3	0	8	5	4	0	17658.8089	-0.005
9	6	3	0	9	5	4	0	17653.9782	-0.002
10	6	4	0	10	5	5	0	17647.3282	0.003
11	6	5	0	11	5	6	0	17638.4121	0.001
12	6	6	0	12	5	7	0	17626.7365	0.001
13	6	7	0	13	5	8	0	17611.7154	0.001
14	6	8	0	14	5	9	0	17592.6473	0.002
15	6	9	0	15	5	10	0	17568.6801	0.001
16	6	10	0	16	5	11	0	17538.7595	0.001
17	6	11	0	17	5	12	0	17501.5587	0.001
18	6	12	0	18	5	13	0	17455.3947	0.002
19	6	13	0	19	5	14	0	17398.1158	0.001
20	6	14	0	20	5	15	0	17326.9990	-0.003
21	6	15	0	21	5	16	0	17238.6408	0.001
22	6	16	0	22	5	17	0	17128.8309	-0.001
8	2	7	0	8	1	8	0	6322.1235	0.003
10	2	9	0	10	1	10	0	7128.6243	0.002
11	2	10	0	11	1	11	0	7593.8482	0.001
16	2	15	0	16	1	16	0	10439.9236	0.000
17	2	16	0	17	1	17	0	11089.9009	0.000
18	2	17	0	18	1	18	0	11758.5791	-0.002
3	3	1	0	3	2	2	0	8034.6527	-0.011
4	3	2	0	4	2	3	0	8042.9212	-0.002
5	3	3	0	5	2	4	0	8059.4427	0.007
6	3	4	0	6	2	5	0	8087.9491	0.001
7	3	5	0	7	2	6	0	8132.5890	-0.002
8	3	6	0	8	2	7	0	8197.7099	0.000

9	3	7	0	9	2	8	0	8287.6808	0.002
10	3	8	0	10	2	9	0	8406.7235	0.001
11	3	9	0	11	2	10	0	8558.7534	-0.001
12	3	10	0	12	2	11	0	8747.2255	-0.001
13	3	11	0	13	2	12	0	8975.0085	-0.002
14	3	12	0	14	2	13	0	9244.2958	-0.002
15	3	13	0	15	2	14	0	9556.5326	0.000
16	3	14	0	16	2	15	0	9912.3600	-0.002
17	3	15	0	17	2	16	0	10311.6268	0.001
18	3	16	0	18	2	17	0	10753.3709	-0.002
20	3	18	0	20	2	19	0	11756.8982	0.004
5	4	2	0	5	3	3	0	11237.4792	-0.003
6	4	3	0	6	3	4	0	11233.0291	-0.003
7	4	4	0	7	3	5	0	11227.1568	0.000
8	4	5	0	8	3	6	0	11220.3464	0.000
9	4	6	0	9	3	7	0	11213.6318	0.001
10	4	7	0	10	3	8	0	11208.6600	-0.003
11	4	8	0	11	3	9	0	11207.7623	0.000
12	4	9	0	12	3	10	0	11213.9037	0.000
13	4	10	0	13	3	11	0	11230.6562	0.001
14	4	11	0	14	3	12	0	11262.0601	0.000
15	4	12	0	15	3	13	0	11312.4868	0.000
16	4	13	0	16	3	14	0	11386.4473	-0.002
17	4	14	0	17	3	15	0	11488.4158	-0.001
18	4	15	0	18	3	16	0	11622.6173	-0.001
5	5	1	0	5	4	2	0	14452.4550	-0.012
6	5	2	0	6	4	3	0	14449.7700	0.001
7	5	3	0	7	4	4	0	14445.5144	0.001
8	5	4	0	8	4	5	0	14439.2395	0.000
9	5	5	0	9	4	6	0	14430.4876	0.000
10	5	6	0	10	4	7	0	14418.8373	0.000
11	5	7	0	11	4	8	0	14403.9674	0.001
12	5	8	0	12	4	9	0	14385.7275	0.001
13	5	9	0	13	4	10	0	14364.2264	0.000
14	5	10	0	14	4	11	0	14339.9191	0.000
15	5	11	0	15	4	12	0	14313.6982	0.000
16	5	12	0	16	4	13	0	14286.9700	0.000
17	5	13	0	17	4	14	0	14261.7159	0.001
18	5	14	0	18	4	15	0	14240.5133	0.000
19	5	15	0	19	4	16	0	14226.5337	0.000
20	5	16	0	20	4	17	0	14223.4773	0.002

21	5	17	0	21	4	18	0	14235.4758	0.001
7	6	2	0	7	5	3	0	17662.1834	0.001
9	6	4	0	9	5	5	0	17654.0094	0.005
10	6	5	0	10	5	6	0	17647.4021	0.003
11	6	6	0	11	5	7	0	17638.6062	0.000
12	6	7	0	12	5	8	0	17627.2082	0.002
13	6	8	0	13	5	9	0	17612.7646	0.000
14	6	9	0	14	5	10	0	17594.8427	-0.001
15	6	10	0	15	5	11	0	17573.0292	0.001
16	6	11	0	16	5	12	0	17546.9608	0.002
17	6	12	0	17	5	13	0	17516.3773	0.001
18	6	13	0	18	5	14	0	17481.1718	0.001
19	6	14	0	19	5	15	0	17441.4509	0.001
20	6	15	0	20	5	16	0	17397.6118	0.002
21	6	16	0	21	5	17	0	17350.4110	0.001
22	6	17	0	22	5	18	0	17301.0437	0.000
23	6	18	0	23	5	19	0	17251.1721	-0.031
24	6	19	0	24	5	20	0	17203.1265	0.002
25	6	20	0	25	5	21	0	17159.6017	0.001
27	6	22	0	27	5	23	0	17100.0692	0.003
28	6	23	0	28	5	24	0	17092.1227	0.007
17	1	16	1	17	0	17	1	10367.9817	-0.001
4	3	1	1	4	2	2	1	7992.0618	0.000
5	3	2	1	5	2	3	1	7950.4018	-0.001
6	3	3	1	6	2	4	1	7880.7707	0.001
7	3	4	1	7	2	5	1	7776.4472	0.001
8	3	5	1	8	2	6	1	7633.6000	0.001
9	3	6	1	9	2	7	1	7452.8110	0.000
10	3	7	1	10	2	8	1	7239.9046	-0.001
11	3	8	1	11	2	9	1	7005.8409	0.002
12	3	9	1	12	2	10	1	6765.8110	0.001
14	3	11	1	14	2	12	1	6341.2421	0.000
15	3	12	1	15	2	13	1	6194.8397	0.000
16	3	13	1	16	2	14	1	6115.8378	0.000
17	3	14	1	17	2	15	1	6118.9418	0.000
19	3	16	1	19	2	17	1	6416.6100	0.000
5	4	1	1	5	3	2	1	11227.1854	-0.002
6	4	2	1	6	3	3	1	11218.7835	0.000
7	4	3	1	7	3	4	1	11204.1208	-0.002
8	4	4	1	8	3	5	1	11180.0297	-0.001
9	4	5	1	9	3	6	1	11142.3282	0.000

10	4	6	1	10	3	7	1	11085.7846	0.000
12	4	8	1	12	3	9	1	10891.1611	0.000
13	4	9	1	13	3	10	1	10740.2745	-0.001
14	4	10	1	14	3	11	1	10547.0158	0.002
15	4	11	1	15	3	12	1	10309.8555	0.001
16	4	12	1	16	3	13	1	10031.6417	0.001
17	4	13	1	17	3	14	1	9720.3437	0.002
18	4	14	1	18	3	15	1	9389.0308	0.001
19	4	15	1	19	3	16	1	9055.0722	0.002
20	4	16	1	20	3	17	1	8738.7406	0.001
6	5	1	1	6	4	2	1	14439.0516	-0.001
7	5	2	1	7	4	3	1	14434.6255	0.000
8	5	3	1	8	4	4	1	14427.8932	0.001
9	5	4	1	9	4	5	1	14418.0491	-0.001
10	5	5	1	10	4	6	1	14404.0646	-0.001
11	5	6	1	11	4	7	1	14384.5927	0.000
12	5	7	1	12	4	8	1	14357.8676	0.001
13	5	8	1	13	4	9	1	14321.5922	0.011
14	5	9	1	14	4	10	1	14272.7530	-0.001
15	5	10	1	15	4	11	1	14207.6000	0.000
16	5	11	1	16	4	12	1	14121.4625	0.001
17	5	12	1	17	4	13	1	14008.8420	0.002
18	5	13	1	18	4	14	1	13863.6304	0.000
19	5	14	1	19	4	15	1	13679.6366	-0.005
20	5	15	1	20	4	16	1	13451.4632	-0.001
21	5	16	1	21	4	17	1	13175.6727	0.000
6	6	0	1	6	5	1	1	17651.4395	-0.001
6	6	1	1	6	5	2	1	17651.4395	-0.001
7	6	1	1	7	5	2	1	17649.1855	-0.001
7	6	2	1	7	5	3	1	17649.1855	-0.002
8	6	2	1	8	5	3	1	17645.8087	0.002
8	6	3	1	8	5	4	1	17645.8087	-0.005
9	6	3	1	9	5	4	1	17640.9723	-0.002
10	6	4	1	10	5	5	1	17634.3119	-0.001
11	6	5	1	11	5	6	1	17625.3907	-0.001
12	6	6	1	12	5	7	1	17613.7072	-0.001
13	6	7	1	13	5	8	1	17598.6771	0.000
14	6	8	1	14	5	9	1	17579.5973	0.000
15	6	9	1	15	5	10	1	17555.6163	0.000
16	6	10	1	16	5	11	1	17525.6799	0.000
17	6	11	1	17	5	12	1	17488.4600	0.001

18	6	12	1	18	5	13	1	17442.2663	0.000
19	6	13	1	19	5	14	1	17384.9500	-0.002
20	6	14	1	20	5	15	1	17313.7936	0.002
21	6	15	1	21	5	16	1	17225.3612	-0.003
8	2	7	1	8	1	8	1	6317.9745	0.004
12	2	11	1	12	1	12	1	8093.8367	0.002
16	2	15	1	16	1	16	1	10431.9591	0.000
18	2	17	1	18	1	18	1	11751.8037	-0.003
5	3	3	1	5	2	4	1	8053.5275	-0.001
6	3	4	1	6	2	5	1	8082.0632	0.000
7	3	5	1	7	2	6	1	8126.7343	0.000
8	3	6	1	8	2	7	1	8191.8845	0.001
9	3	7	1	9	2	8	1	8281.8782	0.002
10	3	8	1	10	2	9	1	8400.9277	0.000
11	3	9	1	11	2	10	1	8552.9357	0.000
12	3	10	1	12	2	11	1	8741.3287	-0.002
13	3	11	1	13	2	12	1	8968.9282	0.001
14	3	12	1	14	2	13	1	9237.6974	0.002
15	3	13	1	15	2	14	1	9546.0755	0.001
16	3	14	1	16	2	15	1	9909.4791	0.001
17	3	15	1	17	2	16	1	10307.3994	-0.005
18	3	16	1	18	2	17	1	10748.9890	0.001
5	4	2	1	5	3	3	1	11229.1841	-0.001
6	4	3	1	6	3	4	1	11224.7332	-0.001
7	4	4	1	7	3	5	1	11218.8586	-0.001
8	4	5	1	8	3	6	1	11212.0546	0.000
9	4	6	1	9	3	7	1	11205.3481	-0.001
10	4	7	1	10	3	8	1	11200.3992	0.000
11	4	8	1	11	3	9	1	11199.5266	0.000
12	4	9	1	12	3	10	1	11205.7086	0.000
13	4	10	1	13	3	11	1	11222.5100	0.000
14	4	11	1	14	3	12	1	11253.9700	-0.001
15	4	12	1	15	3	13	1	11304.4476	-0.001
16	4	13	1	16	3	14	1	11378.4393	-0.001
17	4	14	1	17	3	15	1	11480.3773	-0.001
18	4	15	1	18	3	16	1	11614.4220	0.000
19	4	16	1	19	3	17	1	11784.1738	0.001
6	5	2	1	6	4	3	1	14439.1150	-0.001
7	5	3	1	7	4	4	1	14434.8563	0.000
8	5	4	1	8	4	5	1	14428.5793	0.000
9	5	5	1	9	4	6	1	14419.8249	0.002

10	5	6	1	10	4	7	1	14408.1694	0.001
11	5	7	1	11	4	8	1	14393.2945	0.000
12	5	8	1	12	4	9	1	14375.0537	0.000
13	5	9	1	13	4	10	1	14353.5546	0.000
14	5	10	1	14	4	11	1	14329.2564	0.001
15	5	11	1	15	4	12	1	14303.0477	-0.002
16	5	12	1	16	4	13	1	14276.3471	0.000
17	5	13	1	17	4	14	1	14251.1273	-0.003
18	5	14	1	18	4	15	1	14229.9819	0.000
19	5	15	1	19	4	16	1	14216.0654	-0.001
20	5	16	1	20	4	17	1	14213.0754	-0.002
21	5	17	1	21	4	18	1	14225.1327	-0.003
22	5	18	1	22	4	19	1	14256.6527	0.000
9	6	4	1	9	5	5	1	17640.9998	0.001
10	6	5	1	10	5	6	1	17634.3888	0.002
11	6	6	1	11	5	7	1	17625.5866	0.000
12	6	7	1	12	5	8	1	17614.1807	0.001
13	6	8	1	13	5	9	1	17599.7299	0.000
14	6	9	1	14	5	10	1	17581.8018	0.002
15	6	10	1	15	5	11	1	17559.9773	0.002
16	6	11	1	16	5	12	1	17533.8987	0.000
17	6	12	1	17	5	13	1	17503.3082	0.000
18	6	13	1	18	5	14	1	17468.1001	0.002
19	6	14	1	19	5	15	1	17428.3783	0.001
20	6	15	1	20	5	16	1	17384.5500	0.006
21	6	16	1	21	5	17	1	17337.3602	0.001
22	6	17	1	22	5	18	1	17288.0172	-0.003
23	6	18	1	23	5	19	1	17238.2229	0.001
8	2	6	1	7	1	6	0	15790.9851	0.001
9	2	7	1	8	1	7	0	16639.5500	0.001
5	5	1	0	4	4	1	1	14041.6104	-0.002
5	3	2	1	4	2	2	0	16947.1413	-0.002
8	5	4	0	7	4	4	1	16591.9054	0.000
8	5	3	0	7	4	3	1	16591.6718	-0.009
7	2	6	1	6	1	6	0	16313.6772	0.002
4	3	2	1	3	2	2	0	16126.2068	0.000
4	3	1	1	3	2	1	0	16111.5000	-0.002
9	1	8	1	8	0	8	0	16106.3985	0.007
6	3	3	1	5	2	3	0	17765.5282	-0.001
6	3	4	1	5	2	4	0	17864.6018	-0.003
5	3	3	1	4	2	3	0	16990.5542	-0.001

3	3	1	1	2	2	1	0	15268.3137	0.000
3	3	0	1	2	2	0	0	15265.3345	-0.004
3	3	1	1	2	2	1	0	15268.3137	0.000
3	3	0	1	2	2	0	0	15265.3345	-0.004
6	5	2	0	5	4	2	1	14892.4215	0.007
6	5	1	0	5	4	1	1	14892.3995	-0.002
6	2	4	1	5	1	4	0	14216.6400	0.001
5	2	4	1	4	1	4	0	14166.2718	0.003
15	2	13	0	14	1	13	1	13733.9510	0.004
9	4	6	0	8	3	6	1	14221.5318	-0.001
9	5	4	0	8	4	4	1	17439.3012	0.003
9	5	5	0	8	4	5	1	17439.9692	0.002
7	5	2	0	6	4	2	1	15742.5482	-0.001
7	2	5	1	6	1	5	0	14987.6207	0.003

[a] Observed
[b] Observed minus calculated

Table S4: Measured rotational transitions of the two sub-state of xanthene.

J'	K'_a	K'_c	J''	K''_a	K''_c	obs.^{a}	obs. - calc^b
14	1	14	13	0	13	10246.6229	0.000
14	0	14	13	1	13	10228.7177	0.001
18	1	18	17	0	17	13061.8504	-0.003
18	0	18	17	1	17	13059.9175	-0.001
13	1	12	12	2	11	9908.3650	0.001
14	2	12	13	3	11	9857.1844	-0.003
19	1	19	18	0	18	13767.3110	0.000
19	0	19	18	1	18	13766.2183	-0.001
17	0	17	16	1	16	12353.3227	-0.001
17	1	17	16	0	16	12356.7327	0.001
8	4	5	7	3	4	13846.6822	0.000
5	5	1	4	4	0	13562.0800	-0.001
5	5	0	4	4	1	13562.1001	-0.001
9	3	6	8	2	7	13492.6567	-0.002
8	4	4	7	3	5	13945.6837	0.000
18	1	17	17	2	16	13738.4355	0.001
18	2	17	17	1	16	13811.0382	-0.001
7	3	5	6	2	4	10631.2107	0.002
4	4	1	3	3	0	10639.2226	0.000

4	4	0	3	3	1	10639.6883	-0.001
12	2	11	11	1	10	10056.6418	0.000
6	3	3	5	2	4	10319.4235	0.000
13	2	12	12	1	11	10603.0494	0.001
6	5	2	5	4	1	14382.6186	0.001
7	5	3	6	4	2	15201.7050	0.000
7	5	2	6	4	3	15202.5814	0.000
8	5	4	7	4	3	16017.7777	0.000
8	5	3	7	4	4	16021.0005	0.000
9	5	5	8	4	4	16828.3009	0.000
9	5	4	8	4	5	16837.9727	0.000
10	5	6	9	4	5	17629.1963	0.000
10	5	5	9	4	6	17654.2659	0.000
11	5	7	10	4	6	18414.0745	-0.001
11	5	6	10	4	7	18472.1073	-0.001
9	4	6	8	3	5	14584.1195	0.000
9	4	5	8	3	6	14799.0464	0.000
10	4	7	9	3	6	15264.8182	0.000
10	4	6	9	3	7	15683.9878	0.001
11	4	8	10	3	7	15871.1991	0.001
11	4	7	10	3	8	16619.4682	0.001
12	4	9	11	3	8	16390.8363	0.001
12	4	8	11	3	9	17630.0006	0.002
13	4	10	12	3	9	16820.0459	0.001
14	4	11	13	3	10	17164.3345	0.001
7	7	0	6	6	1	19407.2585	0.000
7	7	1	6	6	0	19407.2585	0.000
13	4	9	12	3	10	18744.7532	0.001
10	6	5	9	5	4	19760.9282	0.000
12	5	7	11	4	8	19295.9654	-0.001
26	1	26	25	0	25	18708.2213	-0.002
26	0	26	25	1	25	18708.2205	0.015
24	1	24	23	0	23	17296.3679	-0.001
24	0	24	23	1	23	17296.3096	-0.001
25	1	25	24	0	24	18002.2884	-0.003
25	0	25	24	1	24	18002.2573	-0.002
22	1	22	21	0	21	15884.5790	-0.001
22	0	22	21	1	21	15884.3882	-0.001
21	0	21	20	1	20	15178.3954	-0.001
21	1	21	20	0	20	15178.7377	-0.001
23	0	23	22	1	22	16590.3551	-0.001

23	1	23	22	0	22	16590.4609	-0.001
7	6	1	6	5	2	17305.5036	0.001
7	6	2	6	5	1	17305.4940	-0.001
6	6	0	5	5	1	16484.6840	0.000
6	6	1	5	5	0	16484.6840	0.000
21	1	20	20	2	19	15881.3517	0.001
18	3	16	17	2	15	15029.1282	0.000
10	7	3	10	6	4	13629.1957	0.000
10	7	4	10	6	5	13629.2488	0.009
9	7	2	9	6	3	13640.8627	0.011
9	7	3	9	6	4	13640.8640	0.001
8	7	1	8	6	2	13649.3208	0.001
8	7	2	8	6	3	13649.3208	-0.001
7	7	1	7	6	2	13655.2540	-0.001
7	7	0	7	6	1	13655.2540	-0.001
24	2	22	23	3	21	18690.8490	-0.001
21	4	18	20	3	17	18685.1318	-0.001
23	3	20	22	4	19	18140.5154	0.001
23	3	21	22	2	20	18085.0144	-0.001
24	2	23	23	1	22	18006.9601	-0.001
18	4	15	17	3	14	18005.3804	0.001
24	1	23	23	2	22	18003.7427	-0.001
12	3	9	11	2	10	17940.8882	0.000
10	9	2	10	8	3	17853.4381	0.000
10	9	1	10	8	2	17853.4381	0.000
11	9	3	11	8	4	17846.7105	0.000
11	9	2	11	8	3	17846.7105	0.000
12	9	4	12	8	5	17837.9666	0.000
12	9	3	12	8	4	17837.9666	0.000
17	4	14	16	3	13	17835.2274	0.001
13	9	5	13	8	6	17826.8295	0.000
13	9	4	13	8	5	17826.8295	0.000
14	9	6	14	8	7	17812.8876	-0.002
14	9	5	14	8	6	17812.8876	0.000
15	9	7	15	8	8	17795.6853	-0.008
15	9	6	15	8	7	17795.6931	0.006
16	9	8	16	8	9	17774.7487	0.001
16	9	7	16	8	8	17774.7286	0.000
17	9	9	17	8	10	17749.5148	0.000
17	9	8	17	8	9	17749.4629	0.000
18	9	10	18	8	11	17719.4072	0.000

18	9	9	18	8	10	17719.2749	0.000
19	9	11	19	8	12	17683.7923	0.000
19	9	10	19	8	11	17683.4732	0.000
16	4	13	15	3	12	17653.1508	0.000
20	9	12	20	8	13	17641.9936	0.001
21	9	13	21	8	14	17593.2995	0.000
22	9	14	22	8	15	17536.9953	0.001
22	9	13	22	8	14	17533.6627	0.000
23	9	15	23	8	16	17472.3918	0.000
23	9	14	23	8	15	17465.7023	-0.001
15	4	12	14	3	11	17436.2995	0.000
22	3	20	21	2	19	17417.8173	-0.001
24	4	20	23	5	19	17589.3454	-0.001
14	8	6	14	7	7	15684.0874	0.000
22	3	19	21	4	18	17165.4889	-0.001
21	3	19	20	2	18	16769.8645	-0.001
22	2	21	21	1	20	16599.2045	-0.001
22	1	21	21	2	20	16589.8279	-0.002
21	2	19	20	3	18	16474.0020	0.000
11	3	8	10	2	9	16282.3864	0.001
20	3	18	19	2	17	16149.1850	0.000
21	3	18	20	4	17	16103.3730	0.001
21	2	20	20	1	19	15897.1950	-0.001
9	8	2	9	7	3	15751.4899	0.000
9	8	1	9	7	2	15751.4899	0.000
10	8	3	10	7	4	15744.0537	-0.001
10	8	2	10	7	3	15744.0537	0.000
12	8	5	12	7	6	15721.2432	0.001
12	8	4	12	7	5	15721.2322	-0.001
11	8	4	11	7	5	15734.1399	-0.001
11	8	3	11	7	4	15734.1399	0.001
13	8	6	13	7	7	15704.8145	0.020
13	8	5	13	7	6	15704.7431	-0.021
13	7	6	13	6	7	13566.8177	0.000
12	7	5	12	6	6	13593.1590	0.000
12	7	6	12	6	7	13593.5921	-0.001
11	7	4	11	6	5	13613.5944	0.000
11	7	5	11	6	6	13613.7418	0.001
24	9	15	24	8	16	17385.9673	0.000
23	2	22	22	1	21	17302.6404	-0.001
23	1	22	22	2	21	17297.1318	0.000

25	9	16	25	8	17	17291.9759	0.000
23	4	19	22	5	18	16154.0727	0.000
24	8	17	24	7	18	15110.1777	0.000
24	8	16	24	7	17	14956.6801	0.000
20	3	17	19	4	16	14946.2419	0.000
10	3	7	9	2	8	14803.4123	0.001
28	8	21	28	7	22	14771.3533	0.001
25	8	17	25	7	18	14760.6310	0.000
24	3	22	24	2	23	14579.6881	0.002
22	2	21	22	1	22	14566.3618	0.003
22	1	21	22	0	22	14562.9539	0.002
24	2	22	24	1	23	14552.3602	0.001
17	8	9	17	7	10	15588.8851	0.000
17	8	10	17	7	11	15590.3523	0.000
16	8	8	16	7	9	15627.0755	0.000
15	8	8	15	7	9	15658.7217	0.000
14	8	7	14	7	8	15684.1789	0.000
20	2	18	19	3	17	15693.9182	-0.001
18	8	11	18	7	12	15545.9211	-0.002
19	2	17	18	3	16	14877.9337	0.000
23	8	15	23	7	16	15112.1875	0.000
20	1	19	19	2	18	15170.9078	0.000
20	2	19	19	1	18	15197.4741	0.000
23	8	16	23	7	17	15201.1209	0.000
19	3	17	18	2	16	15565.5087	0.000
19	8	12	19	7	13	15493.7064	0.000
19	8	11	19	7	12	15486.8218	0.000
20	8	13	20	7	14	15433.1418	-0.001
20	8	12	20	7	13	15419.2709	0.000
21	8	14	21	7	15	15363.9532	0.000
21	8	13	21	7	14	15337.1654	-0.001
22	8	15	22	7	16	15286.3218	0.000
17	3	15	16	2	14	14548.4895	0.000
19	2	18	18	1	17	14501.3950	0.000
20	1	20	19	0	19	14472.9650	-0.002
20	0	20	19	1	19	14472.3532	0.000
19	1	18	18	2	17	14457.2514	0.000
16	3	14	15	2	13	14126.7908	0.000
18	2	16	17	3	15	14013.5863	0.000
15	3	13	14	2	12	13759.0527	0.000
25	1	24	24	2	23	18709.9583	-0.003

25	2	24	24	1	23	18711.8263	-0.003
24	3	22	23	2	21	18765.3637	-0.002
9	6	4	8	5	3	18944.3499	0.003
9	6	3	8	5	4	18944.5563	0.001
13	5	9	12	4	8	19893.7826	0.001
20	7	14	20	6	15	13195.9288	0.000
19	7	13	19	6	14	13267.1744	0.000
18	7	11	18	6	12	13280.6183	-0.001
18	7	12	18	6	13	13334.8101	0.000
17	7	11	17	6	12	13396.3954	-0.001
14	3	12	13	2	11	13431.5991	0.000
16	7	9	16	6	10	13436.7487	0.000
16	7	10	16	6	11	13450.6487	-0.001
15	7	8	15	6	9	13490.6592	0.000
14	7	7	14	6	8	13533.2306	-0.001
14	7	8	14	6	9	13536.0762	-0.001
5	2	4	4	1	3	6684.8607	0.003
9	0	9	8	1	8	6596.8505	0.001
21	5	16	21	4	17	6497.6610	0.001
17	3	14	17	2	15	6417.9165	0.004
15	3	12	15	2	13	5012.2109	0.001
12	2	10	12	1	11	4877.9126	-0.001
11	2	9	11	1	10	4180.2735	0.000
10	3	7	10	2	8	4067.8055	0.001
12	4	8	12	3	9	6227.6173	-0.003
10	3	8	10	2	9	6198.0072	0.000
8	1	8	7	0	7	6179.7108	0.001
11	2	9	10	3	8	6097.3368	0.001
4	2	3	3	1	2	6091.6009	0.001
9	1	8	8	2	7	6091.3809	0.001
9	3	7	9	2	8	5918.5635	0.001
13	4	9	13	3	10	5906.4916	0.000
8	0	8	7	1	7	5826.8825	0.000
9	2	8	9	1	9	5795.7315	0.000
8	3	6	8	2	7	5698.0141	0.000
14	4	10	14	3	11	5611.5282	-0.001
7	1	7	6	0	6	5559.5545	0.001
7	3	5	7	2	6	5531.9456	-0.001
6	3	4	6	2	5	5413.9931	-0.001
15	4	11	15	3	12	5385.8146	-0.001
7	0	7	6	1	6	5028.0359	0.000

6	3	3	6	2	4	4891.9449	0.001
9	1	8	9	0	9	4766.8809	0.001
7	3	4	7	2	5	4684.0055	0.001
8	3	5	8	2	6	4453.8140	0.001
6	2	5	6	1	6	4384.1231	0.000
5	1	5	4	0	4	4375.5819	0.001
6	0	6	5	1	5	4194.7678	0.001
4	2	3	4	1	4	3732.4679	0.000
5	0	5	4	1	4	3326.6178	0.001
11	1	10	11	0	11	6401.0091	-0.001
11	4	7	11	3	8	6535.0877	-0.002
10	4	6	10	3	7	6799.1385	0.000
12	3	10	12	2	11	6939.8490	0.001
9	4	5	9	3	6	7004.4005	-0.001
10	1	9	9	2	8	7130.6323	0.001
8	4	4	8	3	5	7149.8073	0.001
8	4	5	8	3	6	7332.7191	-0.001
10	0	10	9	1	9	7345.0313	0.001
9	4	6	9	3	7	7347.8882	-0.001
10	4	7	10	3	8	7385.0342	0.000
13	3	11	13	2	12	7399.4811	0.000
11	4	8	11	3	9	7453.2527	0.000
15	2	13	15	1	14	7409.4808	0.001
17	5	12	17	4	13	7668.3119	0.002
13	4	10	13	3	11	7718.5952	-0.001
14	3	12	14	2	13	7912.8222	0.002
14	4	11	14	3	12	7930.9070	-0.001
7	2	6	6	1	5	7723.7970	0.001
3	3	0	2	2	1	7721.3427	0.000
3	3	1	2	2	0	7711.9491	0.000
10	1	10	9	0	9	7486.0769	0.001
9	1	9	8	0	8	6823.0037	0.001
11	0	11	10	1	10	8077.9188	0.001
11	1	10	10	2	9	8115.0709	0.002
11	1	11	10	0	10	8164.0227	0.002
8	2	7	7	1	6	8187.9059	0.000
4	3	2	3	2	1	8511.2895	0.001
4	3	1	3	2	2	8558.7700	0.001
7	5	3	7	4	4	9431.9482	-0.001
7	5	2	7	4	3	9428.9700	0.000
5	3	2	4	2	3	9419.4640	0.000

8	5	4	8	4	5	9415.3541	0.000
8	5	3	8	4	4	9406.5661	0.000
9	5	5	9	4	6	9393.9863	-0.002
16	5	12	16	4	13	9380.4209	0.000
9	5	4	9	4	5	9371.6452	-0.001
10	5	6	10	4	7	9368.7782	-0.002
11	5	7	11	4	8	9342.0152	-0.001
15	5	11	15	4	12	9323.8010	0.000
10	5	5	10	4	6	9318.1662	-0.001
12	5	8	12	4	9	9317.6371	0.000
13	5	9	13	4	10	9301.3580	0.001
14	5	10	14	4	11	9300.5373	-0.001
11	5	6	11	4	7	9237.6755	0.000
12	5	7	12	4	8	9119.2205	0.000
10	2	9	9	1	8	9083.0560	0.001
12	1	11	11	2	10	9039.8084	0.001
13	5	8	13	4	9	8950.3341	-0.001
12	1	12	11	0	11	8852.2746	0.001
12	0	12	11	1	11	8800.5973	0.001
14	5	9	14	4	10	8719.8282	0.000
9	2	8	8	1	7	8634.6391	0.000
17	5	13	17	4	14	9479.5879	0.002
13	0	13	12	1	12	9516.7095	0.001
13	1	13	12	0	12	9547.3014	0.000
11	2	10	10	1	9	9552.2155	0.001
15	6	9	15	5	10	11210.4853	0.000
8	3	6	7	2	5	11197.9673	0.000
16	6	10	16	5	11	11075.4077	-0.001
15	1	14	15	0	15	9516.7608	-0.002
16	4	13	16	3	14	8538.5700	0.000
15	2	13	14	3	12	11010.8786	0.001
15	1	15	14	0	14	10948.5977	0.000
15	0	15	14	1	14	10938.2177	0.000
17	6	11	17	5	12	10892.3697	-0.001
19	2	17	19	1	18	10789.8739	0.001
14	1	13	13	2	12	10729.6149	0.001
18	6	12	18	5	13	10649.1341	0.000
14	6	9	14	5	10	11364.7082	-0.001
13	6	7	13	5	8	11381.4682	-0.002
13	6	8	13	5	9	11408.6426	-0.001
12	6	6	12	5	7	11434.3000	-0.001

12	6	7	12	5	8	11446.7209	0.000
5	4	2	4	3	1	11457.9175	0.000
5	4	1	4	3	2	11461.2071	0.000
11	6	5	11	5	6	11473.0377	-0.001
11	6	6	11	5	7	11478.2686	-0.001
10	6	4	10	5	5	11501.3797	-0.001
10	6	5	10	5	6	11503.3778	0.000
15	1	14	14	2	13	11514.2191	0.000
9	6	3	9	5	4	11521.9361	-0.001
9	6	4	9	5	5	11522.6118	-0.001
8	6	2	8	5	3	11536.5866	-0.001
8	6	3	8	5	4	11536.7821	0.000
20	3	18	20	2	19	11741.5741	-0.010
9	3	7	8	2	6	11690.0441	0.001
15	2	14	14	1	13	11812.4082	0.001
16	2	14	15	3	13	12088.6737	0.001
10	3	8	9	2	7	12115.3314	0.000
6	4	3	5	3	2	12270.6523	0.000
16	1	15	15	2	14	12272.1400	0.001
8	3	5	7	2	6	12327.8232	0.000
6	4	2	5	3	3	12283.8860	0.000
16	2	15	15	1	14	12461.2900	0.000
11	3	9	10	2	8	12485.7500	0.000
12	3	10	11	2	9	12816.2168	0.000
17	1	16	16	2	15	13011.5087	0.000
17	2	15	16	3	14	13087.6300	0.001
7	4	4	6	3	3	13070.5150	0.001
6	3	4	5	2	3	9988.2938	0.000
13	7	7	13	6	8	13567.9772	0.000
6	5	1	5	4	2	14382.7926	0.000

[a] Observed
[b] Observed minus calculated

Table S5: **Principal Axis cartesian coordinates of xanthene from M06-2X/6-311++G(d,p) calculation.**

Center	Atomic	Coordinates (Å)		
Number	Number	X	Y	Z
1	8	0.000000	-1.294589	0.345867
2	6	0.000000	1.581775	0.380200
3	6	-1.243623	0.769578	0.123453
4	6	1.243623	0.769577	0.123452
5	6	-1.178601	-0.620805	0.144526
6	6	1.178601	-0.620804	0.144526
7	6	-2.484491	1.363725	-0.103259
8	6	2.484492	1.363725	-0.103259
9	6	-2.314972	-1.404064	-0.032083
10	6	2.314973	-1.404065	-0.032084
11	6	-3.629348	0.599054	-0.281690
12	6	3.629348	0.599055	-0.281689
13	6	-3.541143	-0.790969	-0.239679
14	6	3.541142	-0.790970	-0.239679
15	1	0.000000	2.478433	-0.245116
16	1	0.000002	1.925433	1.422078
17	1	-2.545601	2.446984	-0.130782
18	1	2.545599	2.446984	-0.130782
19	1	-2.211305	-2.481730	-0.007459
20	1	2.211303	-2.481730	-0.007459
21	1	-4.583702	1.081317	-0.452461
22	1	4.583703	1.081316	-0.452460
23	1	-4.427405	-1.398429	-0.377276
24	1	4.427406	-1.398427	-0.377276

Rotational constants (MHz): A = 2042.0, B = 467.9, C = 387.1

Table S6: **Principal Axis cartesian coordinates of xanthone from M06-2X/6-311++G(d,p) calculation.**

**Center**	**Atomic**	**Coordinates (Å)**		
**Number**	**Number**	**X**	**Y**	**Z**
1	8	0.000009	-1.524570	-0.000040
2	8	-0.000024	2.569816	0.000369
3	6	-0.000017	1.356049	0.000110
4	6	-1.239853	0.552780	-0.000148
5	6	1.239850	0.552790	-0.000150
6	6	-1.176508	-0.840924	0.000114
7	6	1.176523	-0.840915	0.000106
8	6	-2.495339	1.174542	-0.000247
9	6	2.495336	1.174555	-0.000242
10	6	-2.339600	-1.612727	0.000062
11	6	2.339617	-1.612718	0.000059
12	6	-3.651661	0.421520	-0.000074
13	6	3.651660	0.421535	-0.000074
14	6	-3.566671	-0.977388	0.000046
15	6	3.566682	-0.977375	0.000048
16	1	-2.516966	2.257984	-0.000460
17	1	2.516952	2.257996	-0.000450
18	1	-2.250930	-2.691666	0.000026
19	1	2.250955	-2.691657	0.000022
20	1	-4.620089	0.905335	0.000007
21	1	4.620088	0.905349	0.000006
22	1	-4.471989	-1.572721	0.000145
23	1	4.472004	-1.572704	0.000147

Rotational constants (MHz): A = 1471.6, B = 466.8, C = 354.4
